# Predictive value of ABVS and VTIQ parameters for axillary lymph node metastasis in breast cancer

**DOI:** 10.3389/fcell.2026.1635743

**Published:** 2026-04-10

**Authors:** Tao Liu, Hongyuan Shen, Jiahong Li, Ruolan Li, Xiaofang Hong, Yujuan Hu, Shuyue Zhang, Tingwei Chen, Weixiang Liang

**Affiliations:** 1 Department of Ultrasound Medicine, Guangdong Provincial Key Laboratory of Major Obstetric Diseases, Guangzhou, China; 2 Guangdong Provincial Clinical Research Center for Obstetrics and Gynecology, Guangzhou, China; 3 Department of Ultrasound, The Third Affiliated Hospital of Guangzhou Medical University, Guangzhou, China; 4 Department of Radiology, Peking University Cancer Hospital and Institute, Beijing, China; 5 Department of Interventional, Guangzhou Red Cross Hospital, Jinan University, Guangzhou, China

**Keywords:** automated breast volume scanner, axillary lymph node, breast cancer, metastasis burden, virtual touch imaging and quantification

## Abstract

**Background:**

This study aims to investigate the predicting value of Automated Breast Volume Scanner (ABVS) combined with Virtual Touch Imaging and Quantification (VTIQ) for axillary lymph node (ALN) metastasis burden in patients with breast cancer.

**Methods:**

This study retrospectively included patients with breast cancer at the Third Affiliated Hospital of Guangzhou Medical University between June 2021 and August 2023. Patients were categorized into the low metastasis burden group (≤2 metastatic lymph nodes) and the high metastasis burden group (≥3 metastatic lymph nodes) based on ALN metastasis status. Image parameters were measured using two-dimensional ultrasound, ABVS, and VTIQ.

**Results:**

A total of 80 patients were included, with a mean age of 55.61 ± 11.10 years, comprising 43 individuals in the LMB group and 37 in the HMB group. The results of multivariate logistic regression analysis revealed that a maximum diameter (OR = 7.386, 95% CI: 1.140–47.845, P = 0.036), the lotus root sign (OR = 5.292, 95% CI: 1.073–26.107, P = 0.041), and mean shear wave velocity (OR = 1.956, 95% CI: 1.263–3.029, P = 0.003) were independent risk factors for high lymph node metastatic burden in the breast cancer patients. The AUC for the parameters of ABVS and VTIQ in predicting lymph node metastatic burden was 0.872 (95%CI: 0.754–0.950, P < 0.001), with the corresponding sensitivity and specificity of 73.0% and 90.7%, respectively.

**Conclusion:**

The combined utilization of parameters from ABVS and VTIQ might have promising predictive value for high ALN tumor burden in breast cancer patients. However, large-scale, multi-center, prospective studies are needed to further confirm our findings.

## Introduction

Breast cancer, the most prevalent malignant tumour among females, has emerged as a significant health concern affecting women worldwide (Cathcart-Rake et al., 2023). The latest edition of the Global Cancer Burden Report shows that there were 2.26 million newly diagnosed breast cancer cases in 2020, and the disease has become the leading cause of cancer mortality among women (Wilkinson and Gathani, 2022). Axillary lymph nodes (ALNs), being the most common site of metastasis in breast cancer, play a crucial role in guiding subsequent diagnosis and treatment. The precise detection of lymph node metastasis status could affect the management of patients with breast cancer in terms of staging, treatment, and prognosis (Skarping et al., 2021). According to discoveries from the American College of Surgeons Oncology Group Z0011 trial, surgical dissection is now deemed necessary solely for breast cancer patients with three or more metastatic ALNs (Giuliano et al., 2017). This shift underscores the updated focus of evaluating ALN status, which now centers on tumor burden—categorized as low burden (<3 positive ALNs) or high burden (≥3 positive ALNs)—rather than the routine assessment of metastasis or non-metastasis (Li et al., 2019; Liang et al., 2019). Therefore, accurately assessing the ALN burden remains a pressing issue (Chang et al., 2020).

Currently, the most common method for pre-surgery ALN metastasis detection is ultrasound examination (Marino et al., 2020; Q et al., 2020), the ultrasound possesses the advantages of low cost, high availability, and is widely used for biopsy guidance. However, the results are dependent, one study showed that ultrasound only achieved a sensitivity of 52.3% on metastasis detection (Kim et al., 2016). With the maturation of advanced ultrasound technologies such as Automated Breast Volume Scanner (ABVS) and Virtual Touch Tissue Imaging Quantification (VTIQ), these innovations hold the potential to overcome limitations associated with conventional ultrasound lesion observation. AVBS can automatically acquire the volume data of the whole breast, and obtain standardized cross-sectional, sagittal, and coronal imaging information of the breast tissue after three-dimensional reconstruction, to realize the offline analysis and diagnosis of breast lesions (Liang et al., 2019; D'Angelo et al., 2021). The VTIQ technique includes two modes, Virtual Touch Imaging (VTI) and Virtual Touch Quantification (VTQ), which can obtain the elasticity score and shear wave velocity (SWV) to reflect the tissue stiffness information (Wang A. et al., 2022; Sun et al., 2019).

This study aims to explore the predictive value of the combination of ABVS and VTIQ technologies in assessing ALN metastatic burden among women with breast cancer.

## Materials and methods

### Study design and patients

This retrospective study included patients with breast cancer at the Third Affiliated Hospital of Guangzhou Medical University between June 2021 and August 2023. The inclusion criteria were: 1) patients with a confirmed diagnosis of malignant breast tumor based on pathological examination; 2) patients who underwent two-dimensional ultrasound, ABVS, and VTIQ ultrasound examinations before surgery; 3) patients who underwent ALN dissection and subsequent pathological examination during surgery. Exclusion criteria were: 1) incomplete clinical data or poor imaging quality; 2) patients who received chemotherapy, radiotherapy, or other anti-tumor treatments before surgery; 3) patients who are multifocal, have undergone breast augmentation with implants, are postpartum, or are lactating. Included patients were categorized into the low metastatic burden (LMB) group (<3 lymph node metastases) and the high metastatic burden (HMB) group (≥3 lymph node metastases) based on lymph node metastasis results (Liang et al., 2019). The study was approved by the Ethics Committee of The Third Affiliated Hospital of Guangzhou Medical University (approval Number: [2023] No. 335), and all enrolled patients provided written informed consent.

### Image analysis

**FIGURE 1:**
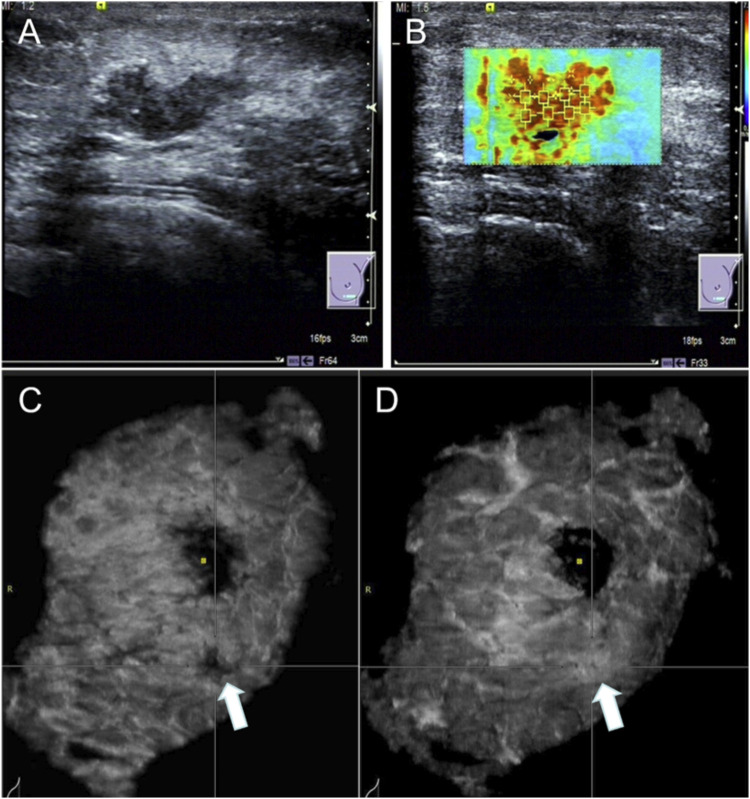
Ultrasound images of a case involving a 63-year-old woman with a lesion measuring 60*50*15 mm in diameter. Pathology results: malignant tumor in the left breast. **(A)** conventional 2D ultrasound image; **(B)** VTIQ average measurement: 6.89 m/s; **(C)** ABVS coronal view showing the mass (arrow); **(D)** “cloud sign” (arrow).

A Simens Acuson S2000 ultrasound scanner with ABVS examination system, 14L5BV free arm probe with 5–14 MHz working frequency, and VTIQ ultrasound software with probe at 7–14 MHz was used. The patient was placed in the supine position with hands raised to have the breasts and axillae fully exposed, after that a sonographer examined the bilateral breast, areola, nipple, and axillae. The ultrasonic presentations and complete imaging data of suspicious masses were documented. Subsequently, the VTIQ ultrasound software was used to measure the shear wave velocity (SWV, in m/s) of the mass area (Figure 1A,B). Six measurement points were taken, one in the region of the highest and lowest velocities, respectively, and the remaining four points were randomly placed, the maximal SWV value (Smax), minimal SWV value (Smin) and mean SWV (Smean) were recorded. Finally, ABVS was applied for a multidirectional examination, and a clear 3D coronal image of the masses was reconstructed. The examination and image interpretation were performed by two sonographers with more than 5 years of experience in ABVS, in case of disagreement a third sonographer was invited to discuss and help to decide the diagnostic results.

### Pathological examination

Pathological examinations of postoperative tissue specimens were completed by the Department of Pathology of the Third Affiliated Hospital of Guangzhou Medical University, and the pathology reports were independently evaluated by two experienced pathologists using a double-blind method. The evaluation of estrogen receptor (ER), progesterone receptor (PR), human epidermal growth factor receptor 2 (HEGFR), and human epidermal growth factor receptor 2 (HERF2) expression status in tumor was completed with Ventana BenchMark XT system (Ventana Medical Systems, Tucson, AZ) The 2018 American Society of Clinical Oncology/College of American Pathologists (ASCO/CAP) guidelines [3] was taken as reference, with≥1% of cancer nuclei considered ER/PR-positive and <1% were considered ER/PR negative; the cut-off value for Ki-67 was 14%; HER-2 expression of 3+ was considered as HER-2 positive, HER-2 expression of + or - was considered as HER-2 negative, and HER-2 expression of 2+ could not be determined, and needed to be further evaluated by fluorescence *in situ* hybridization (FISH). The amplification status of the HER2 gene should be further evaluated by fluorescence *in situ* hybridization (FISH), with positive amplification and negative non-amplification.

### Data collection and definition

Patients’ demographic information and clinical characteristics were collected, including age, age at menarche, family history of breast cancer, type of pathology, presence of vascular invasion, and level of molecular immunologic antigen expression. Blood flow velocity, Adler flow classification (grade 0: no blood flow; grade I: 1-2 spots or little blood flow signal; grade II: 2-3 small vessels or moderate blood flow signal; grade III: 4 small blood vessels and abundant blood flow signal in the lesion area), resistance index (maximal peak flow rate in vasoconstriction-minimal flow rate in diastole)/maximal peak flow rate in vasoconstriction, RI), and peak systolic flow velocity (PSV) were measured and collected by two-dimensional ultrasound.

Imaging features of the mass including the maximum diameter, morphology, margins, microcalcification, convergence sign, lotus root sign, and cloud sign were extracted by the ABVS system. The convergence sign is a specific morphological feature of the breast coronal plane ultrasound images, which is characterized by radial convergence of medium-high echoes and hypoechoic echoes from the periphery of the mass to the inner part of the mass (Xu et al., 2014). The lotus root sign refers to the appearance of round or oval areas of lower echogenicity in the coronal plane of the ABVS of a hyperechoic mass, which are sieve-shaped and resemble a lotus root in cross-section (Tao et al., 2022). The cloud sign is the appearance of unevenly enhanced echoes in the tissue in front of the lesion, resembling a cloud (Figure 1C,D) (Tao et al., 2022).

### Statistical analysis

SPSS 25.0 (IBM, Armonk, NY, United States of America) was used for statistical analysis. The continuous data conforming to the normal distribution were expressed as means ± standard deviation and compared by independent sample t-test. The categorical data were expressed as n (%) and analyzed using the chi-square test. The receiver operating characteristic (ROC) curve was used to determine the optimum cutoff value. Multivariate logistic regression analyses were used to explore risk factors for high ALN metastatic burden in breast cancer patients and to establish the predictive model. The variables with P < 0.05 in the univariable analyses were included in the multivariable regression analyses. The area under the ROC curve (AUC) was employed to assess the diagnostic performance of the model. A two-sided P < 0.05 was considered statistically significant.

## Results

A total of 80 patients were included in this study with a mean age of 55.61 ± 11.10 years, comprising 43 individuals in the LMB group and 37 in the HMB group. Compared with HMB group, patients in the LMB group showed smaller cancer size (37.21% ≤ 2 cm vs. 8.11% ≤ 2 cm. P = 0.002), less frequent lotus root sign (55.81% vs. 89.19%, P = 0.001), fewer occurrences of cloud sign (44.17% vs. 64.57%, P = 0.036), lower rates of positive HER-2 expression (30.23% vs. 62.16%, P = 0.004). The vascular invasion (94.59% vs. 76.74%, P = 0.026) and microcalcification (83.79% vs. 53.49%, P = 0.004) were more prevalent in HMB patients, while the Adler blood flow classification exhibited that HMB patients tend to have high grades (grade II-III: 70.27% vs. 34.88%, P = 0.002). For quantitative parameters, the HMB patients were significantly higher in RI (0.677 ± 0.305 vs. 0.532 ± 0.338, P = 0.030), PSV (25.084 ± 17.724 vs. 16.656 ± 13.173, P = 0.020), and Smean (6.214 ± 1.421 vs. 4.568 ± 1.397, P < 0.001) (Table 1).

**TABLE 1:** Demographic characteristics of patients.

Variable	​	LMB group (n = 43)	HMB group (n = 37)	P
Age (years)	​	55.58 ± 11.08	55.65 ± 11.29	0.979
Age at menarche (years)	​	13.77 ± 1.99	14.03 ± 1.69	0.535
Maximum diameter, n (%)	≤2 cm	16 (37.21)	3 (8.11)	**0.002**
	>2 cm	27 (62.79)	34 (91.89)	​
Family history, n (%)	Yes	5 (11.63)	5 (13.51)	0.799
	No	38 (88.37)	32 (86.49)	​
Tumor shape, n (%)	Irregular	41 (95.35)	32 (86.49)	0.162
	Regular	2 (4.65)	5 (13.51)	​
Smooth margin, n (%)	Yes	2 (4.65)	4 (10.81)	0.297
	No	41 (95.35)	33 (89.19)	​
Convergence sign, n (%)	Yes	16 (37.21)	14 (37.84)	0.954
	No	27 (62.79)	23 (62.16)	​
Lotus root sign, n (%)	Yes	24 (55.81)	33 (89.19)	**0.001**
	No	19 (44.19)	4 (10.81)	​
ER status, n (%)	Positive	36 (83.72)	34 (91.89)	0.271
	Negative	7 (16.28)	3 (8.11)	​
PR status, n (%)	Positive	30 (69.77)	27 (72.97)	0.752
	Negative	13 (30.23)	10 (27.03)	​
Ki-67 status, n (%)	<14%	9 (20.93)	3 (8.11)	0.109
	≥14%	34 (79.07)	34 (91.89)	​
HER-2 status, n (%)	Positive	13 (30.23)	23 (62.16)	**0.004**
	Negative	30 (69.77)	14 (37.84)	​
Adler blood flow classification, n (%)	Grade 0–I	28 (65.12)	11 (29.73)	**0.002**
	Grade II–III	15 (34.88)	26 (70.27)	​
Cloud sign, n (%)	Yes	19 (44.17)	25 (67.57)	**0.036**
	No	24 (55.83)	12 (32.43)	​
Vascular invasion, n (%)	Yes	33 (76.74)	35 (94.59)	**0.026**
	No	10 (23.26)	2 (5.41)	​
Microcalcification, n (%)	Yes	23 (53.49)	31 (83.78)	**0.004**
	No	20 (46.51)	6 (16.22)	​
RI	​	0.532 ± 0.338	0.677 ± 0.305	**0.03**
PSV, m/s	​	16.656 ± 13.173	25.084 ± 17.724	**0.02**
Smax, m/s	​	4.913 ± 1.061	6.704 ± 1.627	​
Smin, m/s	​	4.338 ± 0.719	6.074 ± 1.496	​
Smean, m/s	​	4.568 ± 1.397	6.214 ± 1.421	**<0.001**

PSV: peak systolic flow velocity; RI: resistance index. The indicators marked in bold were statistically calculated in the corresponding groups, and the differences were statistically significant, so they were marked in bold.

**FIGURE 2:**
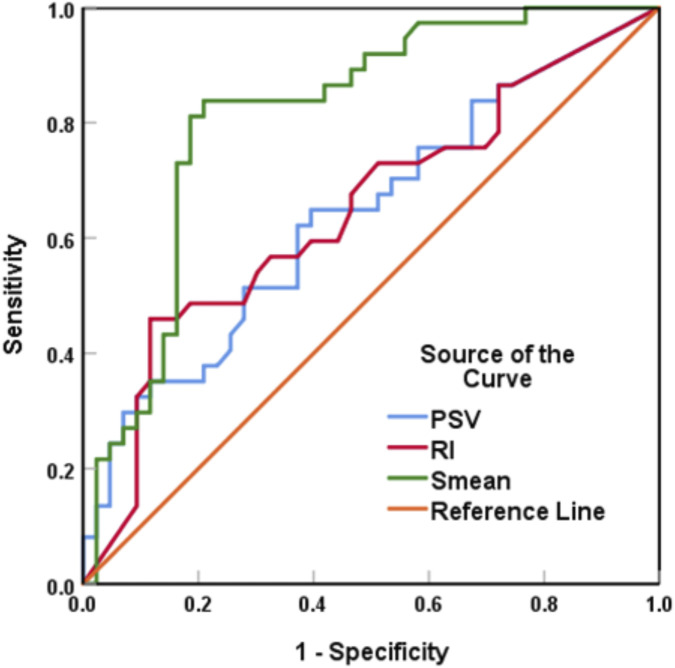
ROC curve analysis of the PSV, RI and Smean for ALN metastasis burden.

According to the results of the ROC curve, the optimized cut-off values for PSV, RI, and Smean were 18.3 m/s, 0.815, and 4.84 m/s, respectively (Figure 2). The results of multivariate binary logistic regression analysis revealed that a maximum diameter (OR = 7.386, 95% CI: 1.140–47.845, P = 0.036), the lotus root sign (OR = 5.292, 95% CI: 1.073–26.107, P = 0.041), and Smean (OR = 1.956, 95% CI: 1.263–3.029, P = 0.003) were independent risk factors for high lymph node metastatic burden in the breast cancer patients (Table 2).

**TABLE 2:** Multivariate logistic regression analysis for high ANL metastatic burden.

Indicators	OR (95%CI)	P value
Vascular invasion	1.255 (0.171–9.166)	0.823
Maximum diameter >2 cm	7.386 (1.140–47.845)	**0.036**
Lotus root sign	5.292 (1.073–26.107)	**0.041**
Cloud sign	1.541 (0.407–5.830)	0.524
Microcalcification	0.668 (0.138–3.239)	0.616
Adler blood flow Classification (Class II–III)	0.797 (0.193–3.291)	0.754
HER-2 status	1.648 (0.441–6.152)	0.458
Smean	1.956 (1.263–3.029)	**0.003**
PSV	0.997 (0.948–1.049)	0.908
RI	3.901 (0.327–46.486)	0.282

PSV: peak systolic flow velocity; RI: resistance index. The indicators marked in bold were statistically calculated in the corresponding groups, and the differences were statistically significant, so they were marked in bold.

**FIGURE 3:**
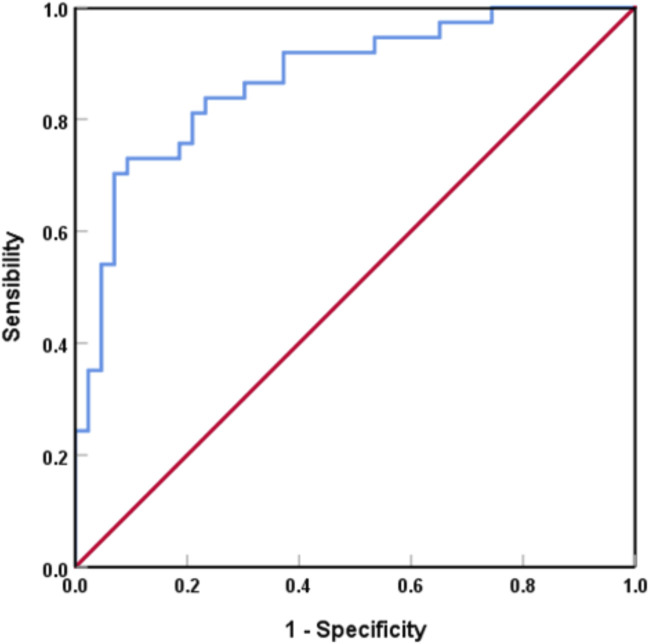
ROC curve analysis of the regression model for ALN metastasis burden.

The AUC for the risk factors in predicting lymph node metastatic burden was 0.872 (95%CI:0.754–0.950, P < 0.001), with a maximum Youden index of 0.637 and corresponding sensitivity and specificity of 73.0% and 90.7%, respectively (Figure 3).

## Discussion

From the parameters of ABVS and VTIQ, a maximum diameter of >2 cm, the presence of a coronal plane lotus root sign, and an elevated Smean value emerged as independent risk factors for a high ALN metastatic burden in breast cancer. These parameters exhibited promising predictive capability for ALN metastasis burden in breast cancer patients.

According to a previous study, VTIQ was used for the prediction of cervical lymph node metastasis in patients with thyroid cancer and exhibited great results, the VTIQ area ratio was the strongest prediction factor (Xu et al., 2016), another study performed by Li et al. developed a risk score model for axillary lymph node metastasis in breast invasive ductal carcinoma where virtual touch tissue imaging played an important role (Fu et al., 2017). In this study, features derived from ABVS and VTIQ techniques were used jointly in the model construction and showed an AUC of 0.872 and our result is in alignment with the previous studies, Wang et al. showed that the combination of ABVS and VTIQ achieved the best performance on malignant and benign breast lesion differential diagnosis, their result exhibited that ABVS-VTIQ performed better than single ABVS or VTIQ technique, although they did not focus on the metastasis of lymph nodes, their research indicated that the clinical potential of ABVS (Wang et al., 2021). These results indicated that the incorporation of both techniques could potentially improve lymph node metastasis diagnostic efficacy.

For specific imaging features, based on this study, we found that the lotus root sign was correlated with high lymph node metastatic load of breast cancer, and the logistic regression model showed a coefficient of 1.666 for the lotus root sign. The lotus root sign is in a sieve-like pattern resembling the cross-section of a lotus root on ABVS coronal imaging of the mass. Previous studies have considered that its formation may be related to the composition of the internal tissue structure of the cancer and the alteration of the internal composition of the breast ducts, and the appearance of the lotus root sign has a certain predictive value for breast malignancy (Tao et al., 2020).

Tumor size as another independent risk factor found by our multivariate regression model was also widely researched in previous studies, Xiong et al. showed maximum diameter was an independent risk factor for ALN metastasis in primary breast cancer, however, their nomogram model only exhibited an AUC of 0.705 in the training set (Xiong et al., 2022); Wang and colleagues further confirmed that minimal diameter was independent risk factor of ALN metastasis in patients with early-stage breast cancer (Wang Q. et al., 2022). However, unlike previous studies, we set the 2 mm threshold for cancer diameter in our protocol, this parameter setting could avoid the adverse influence of extremely large tumor size on our model performance.

The present study also found that the Smean value had a strong correlation with the metastatic load of ALNs. Similar to the results of this study, Zhao et al. (2018) found that ALN metastasis was significantly more frequent in tumors with Smax >6.42 m/s and Smean >5.66 m/s, respectively. The sensitivity, specificity, and accuracy were 85.7%, 54.7%, and 67.0% for Smax; and 59.5%, 79.7%, and 71.7% for Smean. Although in our study, we did not calculate the model performance for each single parameter, our final model yielded a sensitivity of 0.73.0 and specificity of 90.7%, which is higher than in Zhao’s Smena model. While Jiang et al. (2022) concluded that traditional parameters such as SWV are not clinically effective in predicting ALN metastasis, and may even cause some clinical omissions. At present, there is no accurate conclusion about the assessment of VTIQ-related parameters on lymph node metastasis in breast cancer, and a larger number of future studies are still needed to verify it. However, as far as this study is concerned, the efficacy of the prediction model constructed by VTIQ-related parameters is relatively good. Also in this study, after feature selection steps, a total of three features were used to calculate the final score, comparing with previous prediction models, our model possessed the obvious advantages of simplicity and explainability (Wang et al., 2024; Zhang et al., 2024; Wang et al., 2023).

This study had several limitations. First, this is a single-center retrospective study with limited sample size and potential selection bias, further large-scale, multi-center, prospective studies are needed; Second, in this study we only included invasive breast cancer, for other types of breast cancer, the clinical application of our model require further validation. Furthermore, the imaging features evaluated in this study, including the lotus root sign, cloud sign, and convergence sign, were subjectively assessed by ultrasound physicians, thereby introducing an unavoidable observer bias.

The combined utilization of parameters from ABVS and VTIQ might have encouraging predicting value for high ALN tumor burden in breast cancer patients, providing a promising tool for decision-making in breast cancer. Yet, large-scale, multi-center, prospective studies are needed to further confirm our findings.

## Data Availability

The original contributions presented in the study are included in the article/Supplementary Material, further inquiries can be directed to the corresponding authors.
