# Supplementary material for: Predictive value of ABVS and VTIQ parameters for axillary lymph node metastasis in breast cancer
Source: Front Cell Dev Biol. 2026 Apr 10;14:1635743. doi: 10.3389/fcell.2026.1635743 (PMC13106202; doi:10.3389/fcell.2026.1635743)
Supplement: Supplementary file 1 [file DataSheet1.pdf]

Supplementary Table 3. Correlation matrix of candidate predictors

Variable	Diameter	Lotus sign	Smean	Smax	Smin	RI	PSV	Vascular invasion
Diameter	1.00	0.32	0.41	0.38	0.35	0.28	0.33	0.45
Lotus sign	0.32	1.00	0.38	0.35	0.33	0.25	0.22	0.30
Smean	0.41	0.38	1.00	0.89	0.87	0.41	0.44	0.39
Smax	0.38	0.35	0.89	1.00	0.82	0.38	0.40	0.36
Smin	0.35	0.33	0.87	0.82	1.00	0.35	0.37	0.34
RI	0.28	0.25	0.41	0.38	0.35	1.00	0.68	0.29
PSV	0.33	0.22	0.44	0.40	0.37	0.68	1.00	0.31
Vascular invasion	0.45	0.30	0.39	0.36	0.34	0.29	0.31	1.00

Supplementary Table 4. Cut-off values for all ROC analyses

Variable	Optimal cut-off	Sensitivity	Specificity	Youden index
Smean	4.84 m/s	78.4%	79.1%	0.575
Smax	5.92 m/s	81.1%	74.4%	0.555
Smin	4.96 m/s	75.7%	81.4%	0.571
RI	0.62	70.3%	65.1%	0.354
PSV	19.8 cm/s	67.6%	69.8%	0.374

Supplementary Table 5. Model performance at different cut-off thresholds

Cut-off (predicted probability)	Sensitivity	Specificity	PPV	NPV	Clinical application
⩾0.30	91.9%	65.1%	69.4%	90.3%	Screening / rule-out: Minimize false negatives to avoid missing high-risk patients who may benefit from neoadjuvant therapy
⩾0.48 (Youden)	73.0%	90.7%	86.7%	80.0%	Balanced: Optimal trade-off for general clinical use
⩾0.65	51.4%	97.7%	95.0%	70.0%	Confirmation / rule-in: High specificity to confidently identify patients for upfront aggressive therapy
